# Low-Level Laser Therapy Induces Melanoma Tumor Growth by Promoting Angiogenesis

**DOI:** 10.3390/life13020320

**Published:** 2023-01-23

**Authors:** Yi-Yuan Lin, Shin-Yi Lee, Yu-Jung Cheng

**Affiliations:** 1 Department of Exercise and Health Science, National Taipei University of Nursing and Health Sciences, Taipei 112303, Taiwan; 2 General Education Center, China Medical University, Taichung 406, Taiwan; 3 Foreign Language Center, Feng Chia University, Taichung 407, Taiwan; 4 Department of Physical Therapy and Graduate Institute of Rehabilitation Science, China Medical University, Taichung 406, Taiwan; 5 Department of Rehabilitation, China Medical University Hospital, Taichung 404, Taiwan

**Keywords:** melanoma, tumor growth, angiogenesis, low-level laser therapy

## Abstract

The effects of low-level laser therapy (LLLT) on tumor growth are inconsistent. In this study, we investigated the effects of LLLT on melanoma tumor growth and angiogenesis. C57/BL6 mice were challenged with B16F10 melanoma cells and treated with LLLT for 5 consecutive days; untreated mice were used as controls. Tumor weight, angiogenesis, immunohistochemistry, and protein levels were compared between the treated and untreated mice. In an in vitro experiment, B16F10 cells were treated with LLLT. Proteins were extracted and subjected to Western blot analysis for analyzing signaling pathways. Compared with the findings in the untreated mice, tumor weight substantially increased in the treated mice. Both immunohistochemical and Western blot analyses revealed markedly increased levels of CD31, a biomarker of vascular differentiation, in the LLLT group. In B16F10 cells, LLLT considerably induced the phosphorylation of extracellular signal-regulated kinase (ERK), which, in turn, phosphorylated p38 mitogen-activated protein kinase (MAPK). Furthermore, LLLT induced the expression of vascular endothelial growth factor, but not hypoxia-inducible factor-1α, through the ERK/p38 MAKP signaling pathways. Our findings indicate that LLLT induces melanoma tumor growth by promoting angiogenesis. Therefore, it should be avoided in patients with melanoma.

## 1. Introduction

Low-level laser therapy (LLLT), also called photobiomodulation therapy, refers to the use of light (power density < 100 mW/cm^2^; wavelength, 400–980 nm) for physiotherapy. In contrast to other medical laser procedures, this noninvasive therapy exerts its effects through photomodulation rather than through thermal mechanisms. In medicine, LLLT is used to reduce inflammation, relieve pain, accelerate wound healing, and manage soft-tissue injuries [1,2,3,4,5,6,7,8]. However, the safety of LLLT, particularly when targeting areas with tumors, remains debatable. Several in vitro and in vivo studies have reported that LLLT can be harmful in patients receiving treatment for tumors. In urothelial carcinoma (J82) and normal urothelial (HCV29) cells, blue (410 nm), red (635 nm), and infrared (805 nm) light stimulated cell mitosis in vitro; however, the increased irradiation slightly reduced the mitotic rate in mamma adenocarcinoma (MCF7), glioblastoma (U373MG), and gingival mucosa (ZMK1) cells [9]. The exposure (twice) of acute myeloid leukemia (KG-1a) cells to light with an energy density of 20 J/cm^2^ promoted cell growth [10]. However, LLLT with an energy density of 600 J/cm^2^ inhibited the growth of squamous cell carcinoma (VX2) and murine colon carcinoma (CT26) cells in vitro [11]. These findings indicate that the mitogenic effects of light are dependent on the cell type and energy level [12]. At a high dose (1050 J/cm^2^), LLLT (600 nm) markedly increased melanoma tumor volume, blood vessels, and cellular abnormalities compared with the findings in the control group [13]. In an in vivo study that was conducted using nude mice, LLLT promoted the proliferation and angiogenesis of anaplastic thyroid carcinoma cells through the protein kinase B/hypoxia-inducible factor (HIF)-1α pathway [14]. In contrast, LLLT reduced tumor growth in animal studies [15,16] and improved the survival rate in patients with cancer [17].

Several mechanisms have been proposed to explain the effects of LLLT on mitochondria [18,19]. It can alter cell and tissue functions, including the prevention of inflammation-induced apoptosis [20], stimulation of collagen production [21], promotion of DNA synthesis [22], and elevation of ATP levels [23]. In addition, LLLT promotes cell proliferation, vascular endothelial growth factor (VEGF) expression, and angiogenesis [24,25]. Angiogenesis is the growth and development of new capillaries from pre-existing vasculature, and in therapeutic angiogenesis, this phenomenon is employed to alleviate inadequate tissue perfusion [26]. Although LLLT is useful for treating various human diseases, its effects on cellular proliferation remain debatable; it may even induce tumor growth [13].

In tumor cells, the VEGF pathway is the key regulator of angiogenesis and may include angiogenic switches [27]. Tumor cells release angiogenic growth factors, which promote angiogenesis; the activation of angiogenic growth factor signaling pathways, such as the VEGF/VEGF receptor pathway, mediates a network of signaling processes that attract endothelial cells toward the tumor mass, thus promoting endothelial cell growth and migration and angiogenesis [27]. The extracellular signal-regulated kinase (ERK)/p38 mitogen-activated protein kinase (MAPK) pathway is essential for the VEGF-mediated proliferation and migration of cancer cells. In cancer cells, hypoxia upregulates the expression of many angiogenic growth factors, partly through the transcriptional activity of the HIF-1α pathway [28,29].

Melanoma is the least common but the most fatal type of skin cancer [30]. It involves the formation of angiogenic tumors. Angiogenesis facilitates the supply of key nutrients to cancer cells, thus promoting cancer progression and metastasis [31,32]. LLLT, in which the skin is exposed to laser light, may be harmful, particularly for patients with skin cancer. However, the mechanisms underlying the effects of LLLT on tumor growth and angiogenesis are unclear. Therefore, in this study, we investigated the effects of LLLT on tumor growth and angiogenesis in a mouse model of melanoma.

## 2. Materials and Methods

### 2.1. Animal Model and Cell Culture

A total of 20 male C57/BL6 mice (age, 8 weeks) were obtained from the National Laboratory Animal Center, Taipei, Taiwan. The mice were provided with standard laboratory chow and water ad libitum and maintained (in cages) under a 12 h dark/light cycle at 23 °C ± 2 °C in the animal facility of our institute. This study was approved by the Institutional Animal Care and Use Committee of China Medical University (approval number: IACUC# 2017-172). All experiments were performed in accordance with the National Institutes of Health Guide for the Care and Use of Laboratory Animals.

B16F10 melanoma cells were obtained from the Bioresource Collection and Research Center (Hsinchu, Taiwan). The cells were cultured in Dulbecco’s Modified Eagle Medium (DMEM 11995065; Gibco; Thermo Fisher Scientific, Inc., Waltham, MA, USA) supplemented with 10% fetal bovine serum (Hyclone, Logan, UT, USA) and 1% penicillin/streptomycin (Thermo Fisher Scientific, Inc.) at 37 °C in a humidified atmosphere of 5% CO_2_. Before the experiments, the cells were dissociated using 0.25% trypsin–ethylenediaminetetraacetic acid (EDTA; Thermo Fisher Scientific, Inc.). Cell density was evaluated by manual counting using a hemocytometer under a microscope. For animal experiments, B16F10 melanoma cells (5 × 10^5^) were resuspended in 0.1 mL of phosphate-buffered saline and inoculated subcutaneously into the backs of C57/BL6 mice. After inoculation, the mice were randomly divided into LLLT (n = 10) and control (no treatment; n = 10) groups. For in vitro experiments, B16F10 cells were seeded in 10-mm plates at a density of 3 × 10^5^ cells/mL and were incubated for 24 h before LLLT.

### 2.2. Laser Irradiation

After 14 days from the date of tumor cell implantation, the mice were anesthetized with isoflurane. Their fur was removed using a hair removal cream. The tumors were exposed to laser light (AlGaInP diode laser; AM-800; Konftec Co., Taipei, Taiwan) for 5 consecutive days. The probe of the laser device was fixed vertically 30 cm above the mice. Laser irradiation (wavelength, 660 nm) was performed at an output power of 50 mW/cm^2^ for 10 min. The daily average of energy density was 1.91 J/cm^2^. We previously [33] found that 1.91 J/cm^2^ LLLT at 660 nm is sufficient for inducing photomodulation. The mice were anesthetized with isoflurane and euthanized though cervical dislocation 24 h after the last laser irradiation session. Tumor samples were collected for further analysis.

For in vitro experiments, B16F10 cells were irradiated using the same apparatus. The probe of the laser device was fixed vertically 30 cm above the cells inside a laminar flow hood. Laser irradiation (660 nm) was performed at an output power of 50 mW/cm^2^ for 10 min and repeated after 24 h. The daily average of energy density was 1.91 J/cm^2^.

### 2.3. Cell Proliferation Assay

To investigate the effects of LLLT on the proliferation and viability of B16F10 cells, these cells were seeded at a density of 2 × 10^3^ cells/mL in the wells (containing 100 μL of medium) of a 96-well plate. After 24 h incubation, the cells were exposed to LLLT for 10 min; the treatment was repeated after 24 h. Cell viability and proliferation were assessed through an MTT assay [34]. Briefly, after 24 h of second low level LASER irradiation, 10 μL of 5 mg/mL MTT solution was added. After incubation for 4 h at 37 °C, 150 μL DMSO was added to dissolve the purple crystal sediment. The solution was transfer to a new 96-well plate and read with an ELISA reader at 540 nm. The relative cell number was estimated by absorbance value.

### 2.4. Tissue Collection

After the mice were euthanized, the tumors were carefully excised from their backs and weighed. Tumor tissues were divided into three samples that were separately stored at −80 °C for protein analysis, fixed with 10% paraformaldehyde, and embedded in an optimal cutting temperature (O.T.C.) compound and snap-frozen in liquid nitrogen for cryostat sectioning, or fixed with 10% buffered-formalin for paraffin sections.

### 2.5. Immunohistochemical Analysis

To investigate angiogenesis, the tumor tissues were immunohistochemically stained following a previously described method [35]. In brief, tumor tissues that were collected from three mice of each group were soaked in 10% formalin paraformaldehyde. The paraffin-embedded tumor tissues were cut into 4-μm-thick sections. The tissue sections were deparaffinized, hydrated, boiled in Trilogy solution (Cell Marque, Rocklin, CA, USA) for 20 min, and then oxidized in 3% H_2_O_2_. Next, all the tumor sections were stained with CD31/PECAM-1 (ab28364; Abcam, Cambridge, MA, USA) and a rabbit antibody enhancer (D39; Polink-2 Plus HRP Rabbit DAB Detection Kit; GBI LABS). Immunohistochemical analysis was performed using the Polink-2 Plus HRP Rabbit DAB Detection Kit and DAB Quanto Chromogens (TA-060-QHSX and TA-002-QHCX) following the manufacturer’s instructions. CD31-stained areas were observed and photographed using a light microscope (BX43; Olympus, Tokyo, Japan) and were analyzed using ImageJ (National Institutes of Health, Bethesda, MD, USA) to evaluate the density and length to blood vessels in tumor tissues.

### 2.6. Immunofluorescence Analysis

Immunofluorescence analysis was performed as described previously [33]. O.T.C. compound–embedded tissues were cut into 7-µm-thick cryostat sections. Tissue slides were fixed with ice-cold methanol and blocked with 5% normal goat serum. CD31 and collage type IV primary antibodies were used to visualize blood vessels. The secondary antibodies that were used in this experiment were goat anti-rabbit immunoglobulin G (Ig G) conjugated with Alexa Fluor 488 and donkey anti-rat IgG conjugated with Alexa Fluor 594 (Molecular Probes, Eugene, OR, USA). The tissue sections were analyzed and photographed using a fluorescence microscope (BX41M-ESD, Olympus).

### 2.7. Western Blot Analysis

Tumor tissues were homogenized; the cells were lysed in ice-cold buffer (pH 7.5; composition: 25 mM HEPES, 300 mM NaCl, 1.5 mM MgCl_2_, 0.2 mM EDTA, 0.1% Triton X-100, 20 mM β-glycerophosphate, 0.1 mM sodium orthovanadate, 0.5 mM dithiothreitol, 100 g/mL phenylmethylsulfonyl fluoride, and 2 g/mL leupeptin). The protein concentration was measured using the Bradford method (Bio-Rad Laboratories, Hercules, CA, USA). Approximately 40 μg of protein was separated through electrophoresis on a 10% sodium dodecyl sulfate–polyacrylamide gel. Then, the protein bands were transferred onto a polyvinylidene fluoride membrane (pore size, 0.45 μm; Millipore, Bedford, MA, USA) using a transfer apparatus (Bio-Rad Laboratories). Next, the membrane was blocked with 5% nonfat evaporated milk powder dissolved in Tris-buffered saline–Tween 20 buffer (pH 7.6; 25 mM Tris-HCl, 150 mM NaCl, and 20, 0.1% Tween) and was incubated with primary antibodies against CD31, HIF-1α, VEGF, GAPDH, phospho-p38, p38, phospho-ERK, ERK, and β-actin. Table 1 lists the sources of these primary antibodies. After probing with horseradish peroxidase–conjugated secondary antibodies, the protein bands were visualized using an enhanced chemiluminescence reagent (Merck Millipore, Bedford) and analyzed using ImageJ.

### 2.8. Statistical Analysis

The data are presented as the mean ± standard error. Statistical analyses were performed using SPSS Statistics (version 20.0; IBM Corporation, Armonk, NY, USA). Between-group comparisons were performed using a nonparametric Mann–Whitney U test. *p* < 0.05 indicated statistical significance.

## 3. Results

### 3.1. LLLT Enhanced Tumor Progression In Vivo, but Not Cell Proliferation In Vitro

**Figure 1:**
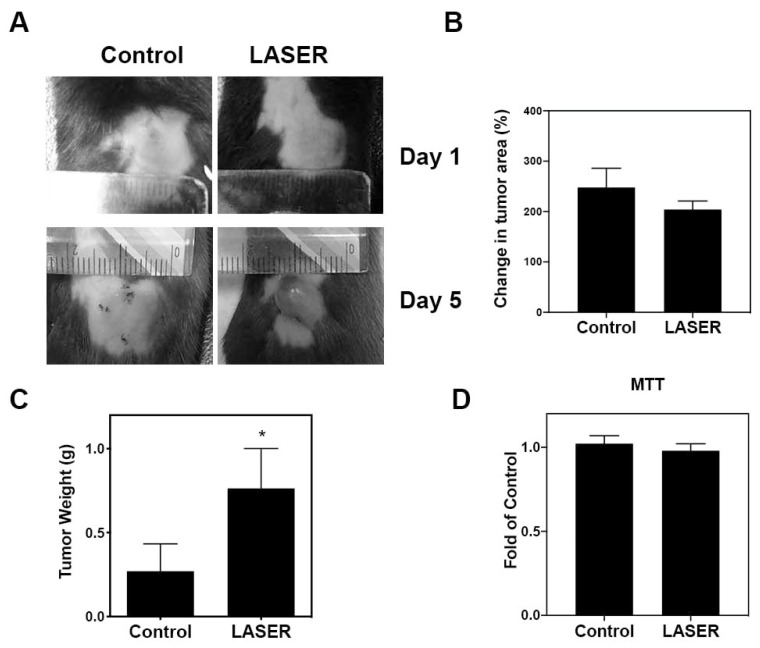
The effects of LLLT on the development of melanoma cell–induced tumors in the experimental mice. (**A**) Representative images of laser-treated (for 5 consecutive days) and untreated tumors. (**B**) Changes in the average tumor area after 5 days. (**C**) Changes in the average tumor weight after 5 days. (**D**) Effects of LLLT on the proliferation of B16-F10 cells assessed through the MTT assay. The data are presented in terms of the mean ± standard error values; * *p* < 0.05 indicates significant differences between the treatment and control groups. LLLT, low-level laser therapy.

To investigate the effects of LLLT on tumor growth, we assessed the progression of B16F10 melanoma in vivo. Figure 1A depicts treated and untreated tumors on Days 1 and 5. Changes in the tumor area at Day 5 from Day 1, and tumor weight at Day 5 are presented using bar graphs (Figure 1B,C). No prominent differences were noted between the treatment and control groups in terms of changes in the tumor area (238.8 ± 42.13 and 204.3 ± 16.56%). By contrast, the tumor weight markedly increased in the treatment group compared with that in the control group (0.2703 ± 0.1628 and 0.7639 ± 0.2373 g). The actual tumor size cannot be measured simply using photographs because the three-dimensional structure of tumors cannot be captured in photographs. To further investigate the effects of LLLT on cell proliferation of B16F10 in vitro, MTT cell viability assays were used. As shown in Figure 1D, LLLT did not enhance the in vitro proliferation of B16F10 cells.

### 3.2. LLLT Promoted Angiogenesis and Expanded CD31-Positive Vascular Area in Tumors

**Figure 2:**
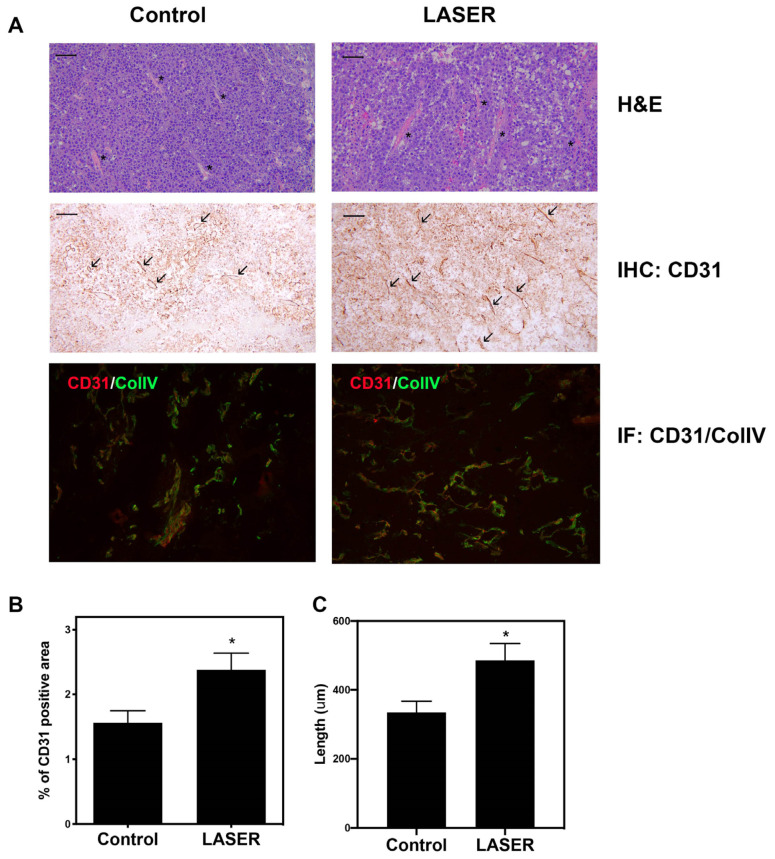
Effects of low-level laser therapy on the angiogenesis of melanoma tumors. The experimental mice were treated with laser light for 5 consecutive days. (**A**) Representative images of tumor tissues that were subjected to hematoxylin–eosin, immunohistochemical, and immunofluorescence staining. The asterisk symbol (*) indicates vascular structure, and the arrowhead indicates CD31-positivity. Bar: 50 μm. (**B**) CD31-positive vascular area that was observed through immunohistochemical staining. (**C**) Vascular length (μm). The data are presented in terms of the mean and standard error values. * *p* < 0.05 indicates significant differences between the treatment and control groups.

Histopathological analysis (hematoxylin–eosin [H&E], immunohistochemical, and immunofluorescence staining) was performed to investigate angiogenesis. H&E staining revealed there were small vessels in the tumors of the control group, and vessels with wide lumen were found in laser light–treated tumors. (Figure 2A). Immunohistochemical staining revealed elevated CD31 expression in laser light–treated tumor tissues. CD31 and collagen type IV dual staining confirmed enhanced vascularization in laser light–treated tumors (Figure 2A). Immunohistochemical staining data were analyzed, and the corresponding bar graphs were constructed (Figure 2B,C). CD31^+^ area and vessel length were both increased in the LASER group.

### 3.3. Angiogenesis-Related Molecules in Tumors

**Figure 3:**
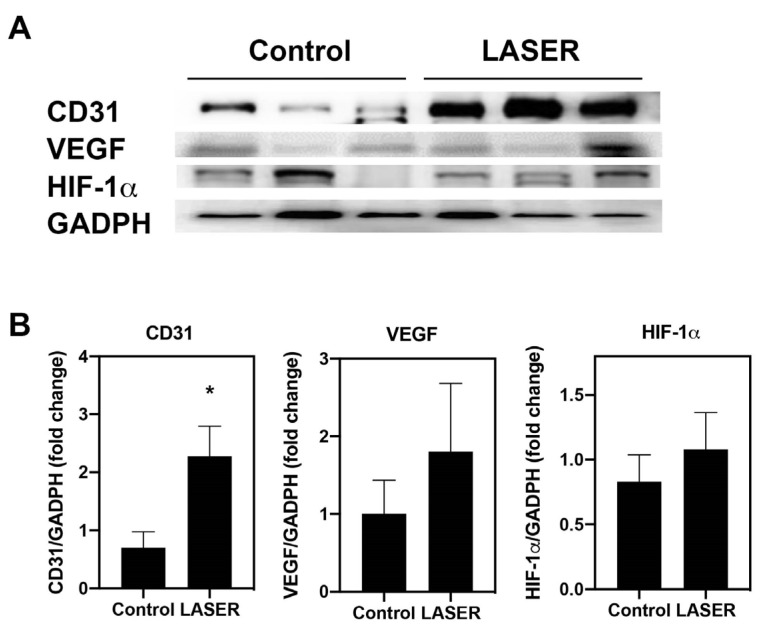
Low-level laser therapy upregulated CD31 expression in melanoma tumors. The experimental mice were treated with laser light for 5 consecutive days. Melanoma tumors were harvested 5 days later. (**A**) Individual mouse melanoma CD31, VEGF, HIF-1α, and GADPH proteins were evaluated through Western blot analysis. (**B**) Results of the relative quantification of CD31, VEGF, and HIF-1α based on GAPDH levels. The data are presented in terms of the mean and standard error values. * *p* < 0.05 indicates significant differences between the treatment and control groups. VEGF, vascular endothelial growth factor. HIF-1α, hypoxia-inducible factor-1α; and GAPDH, glyceraldehyde 3-phosphate dehydrogenase.

Immunohistochemical and immunofluorescence staining in tumor nodules revealed elevated CD31 expression. The protein levels of CD31 was further measured by Western blotting. Similar to the results from immunohistochemical and immunofluorescence staining, the expression level of CD31 in laser light–treated melanoma tumors was 2.2-fold higher than that in the untreated tumors (*p* < 0.05; Figure 3A,B). To understand the effects of LLLT on angiogenic-related signals, the protein levels of HIF-1α and VEGF in the melanoma tumor tissues were measured. However, similar but not significant increases were noted in the expression levels of VEGF and HIF-1α in the treatment and control groups (Figure 3A,B).

### 3.4. LLLT Induced the Phosphorylation of ERK and p38 MAPK in B16F10 Cells

**Figure 4:**
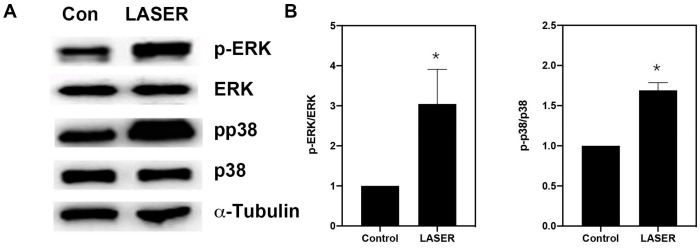

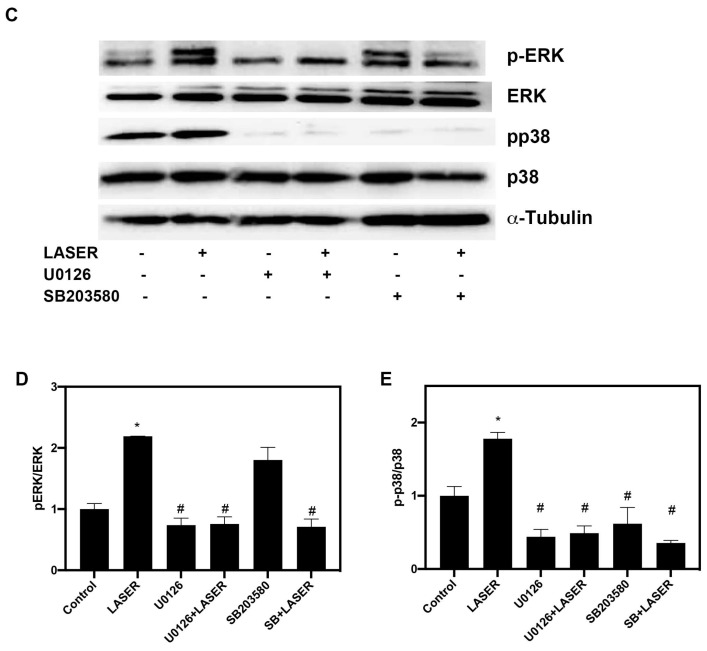
Low-level laser therapy enhanced the phosphorylation of ERK and p38 proteins in B16F10 melanoma cells. The cells were exposed laser light for 10 min for 2 consecutive days. (**A**) Representative Western blot of three independent experiments (n = 3) for phospho-ERK, ERK, phospho-p38, and p38 MAPK proteins. (**B**) The results of the relative quantification of phospho-ERK/ERK and phospho-p38/p38 based on β-actin levels (n = 3). (**C**) B16F10 melanoma cells were exposed to laser light or left untreated. The cells were pretreated with the ERK inhibitor U0126 or the p38 inhibitor SB203580. The levels of phospho-ERK/ERK and phospho-p38/p38 were evaluated through Western blot analysis (n = 3). (**D**) The results of the relative quantification of phospho-ERK/ERK based on β-actin levels. (**E**) The results of the relative quantification of phospho-p38/p38 based on β-actin levels. The data are presented in terms of the mean and standard error values. * *p* < 0.05 and # *p* < 0.05 indicate significant differences between the treatment and control groups. ERK, extracellular signal-regulated kinase.

Western blot analysis revealed substantial increases in the levels of phospho-ERK/ERK and phospho-p38/p38 in B16F10 cells after two sessions of LLLT (Figure 4A,B). We further investigated the phosphorylation of ERK and p38 MAPK in the presence of ERK and p38 inhibitors (U0216 and SB203580, respectively). Pretreatment with U0126 markedly reduced the phosphorylation of ERK from 5.157-fold of the control in the LASER group to 1.25-fold of the control in the U0126-pretreated LASER group. Unlike U0126, SB203580 pretreatment did not change the phosphorylation of ERK (Figure 4C,D). By contrast, both U0126 and SB203580 inhibited the LLLT-induced phosphorylation of p38 MAPK (Figure 4C,E).

### 3.5. LLLT Induced the Expression of VEGF, but Not HIF-1α, through ERK Signaling

**Figure 5:**
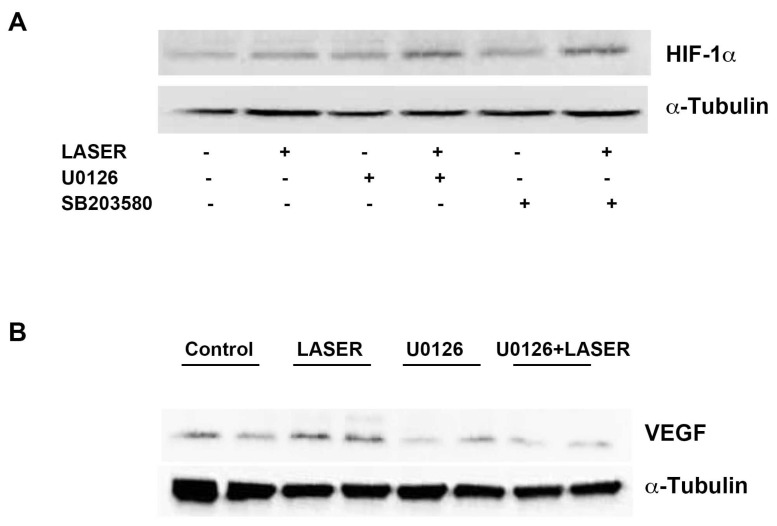
Effects of extracellular signal-regulated kinase and p38 inhibitors on LLLT-induced HIF-1α expression in B16F10 melanoma cells. The cells were pretreated with U0126 or SB203580 and then subjected to LLLT for 10 min for 2 consecutive days. The protein levels of HIF-1α (**A**) and VEGF (**B**) were evaluated through Western blot analysis. The experiments were performed with n = 1 individual per group. LLLT, low-level laser therapy; HIF-1α, hypoxia-inducible factor-1α; and VEGF, vascular endothelial growth factor.

LLLT did not induce prominent changes in the expression levels of HIF-1α and VEGF. We suspected that non-tumor tissues interfered with the results of Western blot analysis. Thus, we further evaluated the expression levels of HIF-1α and VEGF in vitro. To understand the roles of ERK and p38 MAPK in the LLLT-mediated induction of HIF-1α and VEGF expression, B16F10 melanoma cells were pretreated with U0126 and SB203580. The expression levels of HIF-1α and VEGF were measured through Western blot analysis. LLLT increased the expression levels of HIF-1α and VEGF in B16F10 melanoma cells (Figure 5A,B). Compared with the findings that were noted in the control group, HIF-1α expression was upregulated in the treatment group; pretreatment with U0126 or SB203580 did not inhibit this effect. By contrast, pretreatment with U0216 and SB203580 markedly reduced LLLT-induced VEGF expression. These findings indicate that LLLT induces the expression of VEGF, but not HIF-1α, through the ERK/p38 MAPK pathway.

## 4. Discussion

**Figure 6:**
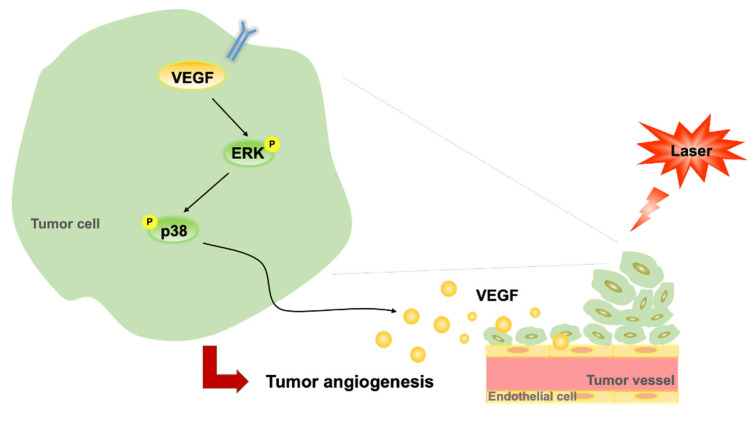
Proposed regulatory pathway through which LLLT promotes VEGF-mediated angiogenesis. LLLT, low-level laser therapy; VEGF, vascular endothelial growth factor; and ERK, extracellular signal-regulated kinase.

In the present study, LLLT did not enhance B16F10 cell proliferation in vitro but increased B16F10 tumor growth through its angiogenic effects in vivo. This therapy induced the phosphorylation of ERK and p38 MAPK and increased the expression levels of VEGF in B16F10 cells. It further induced CD31 expression and enhanced vascularization in subcutaneous tumors. Taken together, our findings suggest that LLLT accelerates tumor progression by promoting angiogenesis and modulating the ERK/p38 MAPK/VEGF signaling pathway (Figure 6).

Melanoma is a highly invasive skin cancer that metastasizes at the early stages [32]. The skin is directly exposed to laser light during LLLT. Therefore, investigating the effect of LLLT on melanoma is crucial. Although LLLT is useful for wound healing, ischemia treatment, and antitumor outcomes, the mitogenic potential of LLLT may increase tumor growth [36]. LLLT at a dose of 150 J/cm^2^ exerts no strong effect on melanoma tumor size, but a higher dose (1050 J/cm^2^) can promote tumor growth with distinct histological features in vivo; this finding indicates that the LLLT dosage differentially affects tumor cell proliferation [13]. In cultured B16F10, our results also showed LLLT did not stimulate cell proliferation, however, we observed that LLLT at a low dose of 1.91 J/cm^2^ upregulated the expression of angiogenesis-related signaling molecules and increased the weight of B16F10 melanoma tumors, which implies that LLLT accelerates the progression and aggressiveness of melanoma through angiogenesis. Frigo et al. also used B16F10 as a melanoma model; however, they inoculated B16F10 cells into BALB/c mice instead of C57BL/6 mice, which are the syngeneic model of B16F10 melanoma and highly susceptible to metastasis. As C57BL/6, but not BALB/c, mice are an immunocompetent syngeneic model for B16F10 melanoma [37], the tumorigenic effects of low-dose LLLT elicited different immune responses in our study. The inhibitory effects that were reported by Frigo et al. might have resulted from the LLLT-induced immune response against melanoma. However, human melanoma may exhibit high immunogenicity through immune evasion [38]. Further studies are needed to clarify the effects of LLLT on immune responses.

Several mechanisms may mediate the effects of LLLT on angiogenesis. LLLT induces VEGF expression in various cell and animal models [25,39,40]. Low-level laser light enhances ERK phosphorylation and VEGF secretion in human granulosa cells [41]. VEGF is a major mediator of angiogenesis by binding with receptor-2 (Flk-1/KDR) and activating the ERK and HIF-1α signaling pathways [42,43,44]. We previously demonstrated that LLLT strongly induces the phosphorylation of VEGF, HIF-1α, and ERK [33]. The expression of both VEGF and ERK is an upstream signal to that of CD31, which is involved in vasculogenesis and angiogenesis [45,46]. Accordingly, LLLT partly promotes angiogenesis by increasing the expression levels of relevant signaling molecules that affect development, reproduction, and wound repair. Angiogenesis is pivotal to cancer development, specifically for tumor growth, progression, and metastasis [47]. The surface of endothelial cells exhibits high levels of CD31, which is a biomarker of vascular differentiation in malignant tissues. CD31 is also a well-known inducer of angiogenesis and is specifically involved in cell–cell communication, which is essential for the maturation of blood endothelial cells [46,48]. Tumors with high CD31 positivity and VEGF positivity indicate early-stage cancer; a higher number of blood vessels implies a higher rate of relapse [46]. In the present study, we found that LLLT increased CD31 levels in tumors, whereas it increased VEGF levels through the ERK/p38 MAPK pathway in B16F10 melanoma cells. Taken together, the results suggest that LLLT accelerates tumor progression by enhancing VEGF expression and thus tumor angiogenesis.

Although most studies have reported similar effects of LLLT on angiogenesis, several studies have indicated the differential effects of LLLT on tumor growth. Ottaviani et al. demonstrated that three LLLT protocols (660 nm, 50 mW/cm^2^, and 3 J/cm^2^; 800 nm, 200 mW/cm^2^, and 6 J/cm^2^; and 970 nm, 200 mW/cm^2^, and 6 J/cm^2^; once daily for 4 days) increased the density of α-smooth muscle actin–positive vessels in tumor samples but decelerated tumor progression [15]. In the present study, we also used the wavelength of 660 nm; however, the fluence is lower than that in the aforementioned study because of a relatively large beam diameter. Studies that were conducted by Da Xing et al. in China have revealed that low-power laser irradiation with high fluence can restrict tumor growth through the photoinactivation of respiratory chain oxidase [49,50]. The inconsistency in these results might be because of the differences in fluence. In addition, different wavelengths, power, optical properties, and cell types may lead to different responses to photochemical stimulation [10,51].

Our study has some limitations. First, the higher expression level of CD31 might not be a good biomarker to tumor progression. Langenkamp and colleagues found the vascular morphology shifts from small vessels without lumen in small B16F10 melanoma to vessels with lager lumen in intermediate/large tumors, and we found similar changes in LASER-irradiated tumors. However, they reported that there is no difference of CD31 mRNA expression in different stages of melanoma [52]. Previous studies have also reported that CD31 is only suitable as a prognostic biomarker for small, but not late stage, laryngeal squamous cell carcinoma [46]. Thus, the LLLT-induced higher protein expression of CD31 in our model might only happen in the early stage of tumor formation. Second, the in vivo and in vitro study findings were inconsistent. For instance, although B16F10 cells and melanoma tumors were irradiated with the same dose, LLLT markedly upregulated the expression of VEGF and HIF-1α in B16F10 cells, but not in melanoma tissues. Whether the degrees of LLLT-mediated photomodulation are similar between in vivo and in vitro experiments must be investigated further. Tumor microenvironments vary across cancer types and are crucial for tumor growth and immune evasion. The differences between in vitro and in vivo findings imply that the mitogenic effects of LLLT on B16F10 cells are not 100% similar to its mitogenic effects on patients with melanoma. Further studies are needed to validate our findings.

## 5. Conclusions

In the present study, LLLT (wavelength, 660 nm; power density, 50 mW/cm^2^; energy density, 1.91 J/cm^2^) increased tumor weight and promoted angiogenesis. Distinct histological features were observed. Thus, LLLT may aggravate melanoma, thereby worsening disease prognosis. Our in vitro and in vivo study findings indicate that LLLT is unsafe for patients with skin cancer, particularly those with melanoma. Therefore, clinicians and physiotherapists must avoid this therapy in patients with skin cancer. 

## Figures and Tables

**Table 1:** Antibodies for Western blot analysis.

ERK	Cell Signaling #9102
p-ERK	Cell Signaling #9101
P38 MAPK	Cell Signaling #9212
p-p38 MAPK	Cell Signaling #9211
CD31	Invitrogen #PA5-16301
HIF-1α	Invitrogen # MA1-516
β-actin	GeneTex #GTX109639

## Data Availability

The datasets generated and analyzed in this study are available from the corresponding author upon reasonable request.
